# Experiences of psychiatrists and support staff providing telemental health services to Indigenous peoples of Northern Quebec

**DOI:** 10.1186/s12913-021-06072-5

**Published:** 2021-01-23

**Authors:** Zhida Shang, Antonia Arnaert, Yvonne Hindle, Zoumanan Debe, Geneviève Côté-Leblanc, Amine Saadi

**Affiliations:** 1 Ingram School of Nursing, McGill University, Montréal, Québec Canada; 2 Centre Intégré Universitaire de Santé et Services Sociaux de l’Ouest-de-L’Île-de-Montréal, Montréal, Québec Canada

**Keywords:** Telemental health, Telepsychiatry, Videoconferencing, Remote consultation, Mental health, Indigenous peoples, Northern Quebec

## Abstract

**Background:**

Due to regional, professional, and resource limitations, access to mental health care for Canada’s Indigenous peoples can be difficult. Telemental health (TMH) offers the opportunity to provide care across vast distances and has been proven to be as effective as face-to-face services. To our knowledge, there has been no qualitative study exploring the experiences of TMH staff serving the Indigenous peoples in Northern Quebec, Canada; which is the purpose of this study.

**Methods:**

Using a qualitative descriptive design, the entire staff of a TMH clinic was recruited, comprising of four psychiatrists and four support staff. Individual semi-structured interviews were conducted through videoconferencing, and results were thematically analyzed.

**Results:**

To address the mental health gap in Northern communities, all psychiatrists believe in the necessity of in-person care and note the synergistic effect of combining in-person care and TMH services. This approach to care allows psychiatrists to maintain both an insider and outsider identity. However, if a patient’s condition requires hospitalization, then the TMH staff face a new set of information sharing and communication challenges with the inpatient staff. TMH staff believe that the provision of culturally sensitive care to Northern patients at the inpatient unit is progressing; however, more work needs to be done. Despite the strong collegial atmosphere within the clinic and collective efforts to provide quality TMH services, all participants express a sense of frustration with the paper-based and scattered documentation system.

**Conclusion:**

The TMH team works in cohesion to offer TMH services to Indigenous peoples; yet, automatization is needed to improve the workflow efficiency within the clinic and collaboration with the Northern clinics. More research is needed on the functioning of TMH teams and the separate but important roles of each team member.

**Supplementary Information:**

The online version contains supplementary material available at 10.1186/s12913-021-06072-5.

## Background

Indigenous peoples around the globe are resilient, and their identity and culture are a fundamental source of strength. However, the high incidence of mental health issues within this population is troubling, which may be attributed to the historical oppression, colonization and the continuous social, political and environmental challenges that they often face [1]. Besides difficulties associated with colonization and geographical remoteness [2], these Indigenous communities are confronted with a high turnover and a lack of qualified mental healthcare providers [3, 4]. To improve access and support local mental health frontline workers, the Canadian government in collaboration with the Cree and Inuit health boards, have created alternative service models: 1) travelling psychiatrists who make periodic visits to the Northern communities, and 2) videoconferencing-based telemental health (TMH) [5]. TMH is defined as the provision of mental or behavioral health services, in real-time, through information and communication technology, involving a wide range of professionals and support staff [6]. Most TMH studies are quantitative, with a comparably fewer qualitative studies [7]. Existing qualitative studies tend to focus on understanding the experiences of a single group, such as patients, psychiatrists or allied healthcare providers (i.e. therapists and nurses) [8–13]. As for the use of TMH in the Indigenous Canadian context, researchers have found that cultural rapport can be built, albeit with some difficulty [14, 15]. To further strengthen this rapport, researchers [15, 16] recommend providers to visit the Indigenous communities, in order to foster an understanding of the local land and the historical, cultural and social context of their patients. Overall, there is a lack of information around the functioning of a comprehensive TMH team, especially the experiences of support staff. To our knowledge, no qualitative study has explored the experiences of both psychiatrists and support staff working in a TMH clinic within the Indigenous Northern Quebec context.

Evidence exists that TMH is an effective care modality for children and adults, with equivalent treatment outcomes to in-person care across a range of mental health disorders and patient populations [17]. Although patients and providers are largely satisfied with TMH as it improves access to care [14], reduces wait and travel times and costs [18], providers report a combination of system, policy and administrative concerns and are often seen as the clinical gatekeepers for implementation and sustainability of these services [9, 19, 20]. Despite advances in videoconferencing software programs, technical issues appear to be the most prevalent [21], and providers have noted video, audio and latency issues, and an inflexible video camera as barriers to patient care [19, 22–25]. For example, providers are often troubled if a technical issue occurs when a patient is discussing an emotional and sensitive topic [23], and have difficulty not accidently interrupting the patient during audio lags [24]. Relatedly, technical issues may prevent providers from seeing facial expressions, tics and tremors [19], which may inherently translate in a decreased sense of therapeutic alliance building [10, 14, 24], with TMH being perceived as impersonal [14, 24]. One study [15] interviewed providers who believed that there are some patient drawbacks using TMH, including less patient engagement, challenges sharing information within the care team and greater inefficiency. As for patient suitability for TMH, providers’ opinions are mixed. In one study [22], providers indicate that all mental health patients can be treated via TMH, and identify patients with anger management issues and agoraphobia as those who best respond to TMH. In addition, some psychiatrists believe that shy or socially anxious patients may be well treated through TMH [15, 25]. Conversely, other providers believe that patients who are emotionally unstable, impulsive or have poor coping skills, and those suffering from dementia, paranoia, visual and/or hearing deficits are not suitable for TMH [10, 14].

Hybrid service models, defined as the combination of in-person and TMH interventions for diagnosis, therapy and monitoring, may synergistically combine the advantages of both modalities, while minimizing the disadvantages [26, 27], such as increasing the number of outpatient consultations, while maintaining patient engagement [28]. From the provider’s perspective, in-person consultations may be cumbersome because of the costs and time associated with travel [19], leading to one study recommending only the initial consultation to be in-person [14]. The provision of culturally sensitive care is key in mental health, but when providing TMH services, cultural misunderstandings may be amplified [29], mainly due to geographical distance, technological issues and unfamiliarity with local languages and customs [30]. Having TMH providers who speak the local language or are of the same cultural background reduces the loss of language nuances, enhances the therapeutic alliance and increases the sense of confidentiality [31, 32]. Overall, there are various challenges present in Western-based technologies such as the difficulty of integrating traditional Indigenous teachings and historical/cultural ways of healing, leading to challenges in maintaining the therapeutic alliance and patient engagement [33]. Thus, the purpose of this study is to explore the experiences of both psychiatrists and support staff working in a TMH clinic that is mandated to provide hybrid services to Indigenous peoples in Northern Quebec, Canada.

## Methods

### Context

Since 2007, the TMH clinic, part of a university-affiliated psychiatric hospital, is mandated by the Quebec provincial government to provide remote clinical consultations to adult patients from various Cree and Inuit communities. Occasionally, this service is also used for medical education, family meetings, legal and administrative tasks. The TMH services are conducted by the four psychiatrists, who provide consultations, assessments, treatment, and follow-ups via videoconferencing. In addition, each psychiatrist travels one week per month to a Northern Indigenous community to provide in-person care. To ensure continuity of care, every psychiatrist is assigned to work with the same Northern community throughout their career as TMH staff, with each covering different communities. As for the clinical consultations, the psychiatrists have according to their individual schedules, the flexibility to start and end the videoconferencing session, through a software program installed on their desktop computers.

All patients from the Northern communities receiving services from the TMH clinic are Indigenous. Patients need to access a room with fixed videoconferencing equipment at a local clinic. The support staff, consisting of a manager, two liaison nurses and a clinical secretary, are responsible for the logistical organization of the TMH services. More specifically, the liaison nurses work with the inpatient staff caring for hospitalized Indigenous patients, relay patient information to the TMH staff, participate in inpatient rounds, arrange family meetings through videoconferencing, and are responsible for discharge planning. The clinical secretary is solely involved in administrative work, with no direct patient care responsibilities. Duties of the clinical secretary include scheduling the psychiatrists’ videoconferencing consultations, receptionist tasks and coordinating medical documentation. The manager oversees the work of all TMH staff, organizes weekly patient care rounds specific to the TMH clinic and communicates any concerns or questions to the higher hospital management.

### Participants and procedure

Ethical approval from the Douglas University Institute Research Ethics Board was granted in 2019. Using a qualitative descriptive design, a purposive sample, consisting of the entire TMH staff was recruited. Prior to the study, there was no relationship between the authors and the participants. A recruitment flyer was disseminated, and the study was presented during one of their weekly staff meetings. The sole inclusion criterion for participant was employment at the TMH clinic. Written consent was obtained prior to the one-time semi-structured interview. The interviews were conducted in English via videoconferencing (ZOOM) by the first and second authors, at a time convenient for the participant and lasted approximately 60–75 min. Some interviews were organized from a private office at the clinic or at home. The second author co-facilitated the interview and made field notes to supplement the audiotapes. The interview guide (Additional file 1) included questions such as: *Tell me about your role within the TMH clinic. Can you explain how a TMH consultation is organized? In your opinion, what are the challenges you currently encounter when organizing and delivering a TMH videoconferencing consultation? What is your experience communicating with local frontline workers in the Northern communities? In your opinion, what are your recommendations to improve the TMH services?* In order to ensure alignment between the study aim and the interview questions, the guide was pilot-tested one month prior to data collection, with a general practitioner (GP) who works closely with the TMH staff and a telehealth coordinator of the university-affiliated psychiatric hospital. Further refinements were made after the first few interviews [34].

### Analysis

All audio-recorded interviews, supplemented with field notes, were transcribed verbatim and manually thematically analyzed using an inductive approach [35, 36]. The data analysis followed the iterative process similar to a qualitative study conducted by the second and fourth author [37], which involves open coding [38] and enhancing the trustworthiness of the study [39] through member checking, data triangulation and reflective journaling. A more detailed description of the data analysis process can be found in the methods section of the previously mentioned study [37]. To respect the anonymity of each participant, no personal identifying information will be presented in the results.

## Results

A total of four psychiatrists, two liaison nurses, one clinical secretary and one manager, representing the four psychiatrists and four support staff working at the TMH clinic, are included in the study. Seven out of the eight participants are of a White-Caucasian background, and one identifies as Latinx. No participants are of an Indigenous background.

### Vacuum of mental healthcare in northern communities

The need for local Northern mental health services is an issue raised by three psychiatrists; whereby two psychiatrists indicate being happy to see their diminished role in return for further local mental health services, as one explains: “I hope they will have their own psychologists and psychiatrists, so they won’t need me. And I am going to be there until I am more detrimental to them and then I will leave”. This lack of services is intensified by the shortage of Northern staff, such as nurses, GPs, social and community workers, and is further aggravated by the high turnover rates that is so common up North.

Similarly, the lack and turnover of community workers and social workers are of concern to some TMH staff, with one psychiatrist giving an example: “We had somebody with borderline personality disorder receiving therapy from the social worker, and of course the social worker went on vacation, and it was a crisis”. Another psychiatrist expressed that more patients don’t show up for their TMH appointments when the local community worker is missing. Overall, working with the Northern staff is an appreciated experience for all the TMH psychiatrists, and a delicate experience for one, describing their unique concerns: “I need to be careful of what I am saying because I can re-trigger traumas in those social workers I’m working with”.

### Synergistic effect of videoconferencing and in-person care

To begin, all psychiatrists believe in the necessity of regular in-person consultations up North and that videoconferencing cannot fully replace the in-person consultations. All psychiatrists state that they enjoyed the in-person consultations, and they have identified various broad advantages, such as the ability to better discuss sensitive issues, build the therapeutic alliance and collaborate with the Northern teams. One psychiatrist highlights the benefits of videoconferencing: “We are talking about sensitive issues like trauma and suicide, so people want to know that the person they are talking to is a person, not someone on a screen”. Relatedly, three psychiatrists state the necessity of having a first contact with the patients in-person, unless it is an emergency first evaluation.

Naturally, the in-person consultations allow the psychiatrists to develop an “insider” role, which enables them to gain a deeper understanding of the Indigenous culture and local community life. All psychiatrists embrace the insider role that they adopt, and state various methods of becoming immersed in the community, such as: participation in sweat lodges and feasts, living in proximity with the community and having casual conversations with community members. This can be summarized by one psychiatrist: “The reality of living in these Northern communities is very different than living in the city, and you have to go and see how things function, which our patients appreciate us knowing”. Overall, all psychiatrists believe that this insider role is a demonstration of allyship, an act that is necessary for the mental health care of Indigenous peoples.

On the other hand, all psychiatrists also believe in the necessity of videoconferencing, with the primary benefit being the ability to see more patients. However, for all psychiatrists, videoconferencing is just one method for them to maintain a concurrent “outsider” identity. This outsider identity is one that is necessary for the field of mental health, as it allows the patients to disclose sensitive information without fear of it leaking out to the community. Therefore, the psychiatrists engage in their insider role to become involved with the community but cannot become too involved or else the patients will be uncomfortable disclosing sensitive information. One psychiatrist summarizes the outsider role as: “People appreciate that I am not from the community, so there is no concern that after my day I am going to get drunk and talk about my clients”. Another psychiatrist explains patients’ perceptions: “Patients feel more comfortable opening up about difficult things because they know that even though we are there recurrently, we are not part of all of those gatherings. That we are able to listen and take the plane and fly away and not gossip or share”. This inherent and complex balance between the two roles during their in-person consultations is a task that the psychiatrists must continuously manage. In a sense, the outsider role is maintained by the videoconferencing, however it is not solely about it, as it can also be maintained by simply not being a permanent member of the community.

As for the videoconference itself, there are still technical limitations. The primary technological issue identified by three psychiatrists is the fact that their desktop computers are outdated. This reduces the speed at which they can run the videoconferencing software, and subsequently a reduction in the efficiency of their TMH services. These technological issues are noted by the manager, who comments: “We need money to purchase new equipment, like a portable computer or desktop, in order to have good quality videoconferences, with assessments, follow ups, team meetings, whatever. And I think being told that we have to wait for new equipment until the old ones die doesn’t work”. Despite these persisting technological issues, there has been gradual improvement over the years, and two psychiatrists noted that the current TMH system is immensely superior to the old fixed-videoconferencing system, where the psychiatrists had to compete for a single, overly large videoconferencing room.

Due to these inherent technological issues, psychiatrists believe that there is a varying acceptability of the videoconferencing technology amongst patient populations and age groups. In general, psychiatrists identify younger Indigenous peoples as more acceptive towards the videoconferencing technology, compared to older patients, who often are hard of hearing, present with an English language barrier and have less technological literacy. Relatedly, another psychiatrist believes that there is a varying acceptability amongst certain mental health conditions, as videoconferencing may be more suitable for patients with depression and anxiety, rather than acute conditions such as psychosis. The psychiatrist further elaborates: “If there is a patient who has a psychotic illness, and there is somebody within the camera, it can tap into the delusional story”.

### Variation of care between inpatient and TMH staff

In the case of a serious clinical presentation, Indigenous patients must be hospitalized at the university-affiliated psychiatric hospital. When the patients are flown down South and admitted, the TMH psychiatrists, with their knowledge on Indigenous mental health, serve in a consultant role in support of the inpatient unit psychiatrists, which is seen by a TMH psychiatrist and liaison nurse as a more backpedalled role. This role may also create some confusion for the admitted patients, as one psychiatrist finds it troubling that patients must go through a variety of different psychiatrists as their mental health provider.

One of the main differences between the TMH and inpatient psychiatrists is about medication dosages, as TMH psychiatrists prefer to prescribe a lower dose. For this issue, a TMH psychiatrist recommends the initiation of once-monthly injections at a local Northern clinic as an alternative, since “patients don’t have to be seen taking drugs at home, [which] are often overcrowded, so they feel less stigma”. In addition to the medication doses, two liaison nurses and three TMH psychiatrists have a different cultural care approach towards the admitted patients. Liaison nurses and psychiatrists believe that their experience in working with Indigenous patients is what makes them different from the inpatient staff. Naturally, all the mentioned participants believe in the importance of communicating with and teaching the inpatient staff.

Although the liaison nurses engage in teaching the inpatient staff, they also play a more direct patient care role within the inpatient unit, in stark contrast to the TMH psychiatrists. The liaison nurses primarily serve as a conduit of information between the TMH clinic and the inpatient unit. In addition, the liaison nurses are experts in cultural care, with one describing the use of humor and open-ended questions as techniques in culturally safe care. One liaison nurse who places particular importance in their care for re-admitted patients, says: “A lot of times they will recognize me. I will joke with them by saying ‘oh you’re back again?’, I think they feel more comfortable because they know somebody”.

### Positive TMH working environment

All participants enjoy working with other TMH staff members, manifested as an intense cooperation. One psychiatrist values the therapeutic value of working with colleagues facing similar conditions, as they enjoy the opportunity to just: “get together and exchanging different strategies, but also just being there and being able to put it out of your chest”. The tight knit collaboration that is present within the TMH clinic is facilitated by the positive and rapid culture of communication. Two psychiatrists, two liaison nurses and the manager all mention about the importance of communication within the TMH clinic. All those participants believe that the physical layout of the TMH clinic is conductive to rapid communication, noting the importance of having their offices close to each other.

The culture of teamwork enables the TMH staff to engage their mandate and deliver culturally sensitive care to the Northern communities. In order to deliver competent TMH care, two psychiatrists emphasize the importance of: listening, acknowledging their own power and always being careful about what they say. One psychiatrist explains: “Patients will sometimes have questions, but the answers will come at right time. But still, sometimes ten years later, I don’t have the answer. It’s very complex and you have to go with the flow, it is what I call a dance”. Nonetheless, one of the psychiatrists comments that in working with Indigenous peoples: “We have to realize that there is a lot that we are powerless to help with, since there is a lot that is beyond the scope of what we can do, and we have to deal with that frustration”. However, the same psychiatrist later adds: “Part of the frustration is necessary because this is the frustration that the patients feel, right? By feeling the frustration, we are getting closer to their experience”.

### Need for Digital transformation

All participants mention that there is a need for a comprehensive and automized documentation system, as the current system is a paper-based one, and each psychiatrist has a different method of writing their own patient notes. The clinical secretary and all liaison nurses and psychiatrists provide specific recommendations towards improving the current documentation system, such as: having an integrated documentation system that is easily accessible at the university hospital and at the Northern clinics, making the documentation system accessible to all professionals and staff involved in the patient’s care and being able to securely access the documentation system from their personal computers. Despite the call for comprehensive automatization throughout the TMH clinic, a partial clinical documentation platform was implemented in early 2019, allowing some TMH psychiatrists working with a select few communities to electronically document their work. The clinical documentation platform is also accessible for staff at the Northern clinics.

Overall, the lack of automatization and the scattered methods of documentation have negative implications for the TMH staff. One of the main consequences is frustration, a feeling that is expressed by two psychiatrists and the clinical secretary. One psychiatrist elaborated: “As a detailed oriented person, I need to have a sense of and to take control of what is going on, which is causing me a lot of time and frustration”. As a result of this concern and frustration, another psychiatrist decided to take matters into their own hands and advocate for these issues with the local health board. However, the psychiatrist described their experience in advocacy: “It is like a dance, because if you argue too personally, it gets crude, but if you are too proactive you will get burnout, yet if you are too passive it is not good either”.

## Discussion

Findings revealed that TMH care is seen by all participants, psychiatrists as well as support staff, as an extended service comparable to conventional in-person mental health care. Although TMH may be suitable for most mental health conditions, it is particularly advantageous for people living in remote communities when provided in conjunction with in-person visits. Paying attention to the cultural factors make TMH effective and rewarding for both the provider and Indigenous patient. However, in terms of sustainability, three points warrant further discussion: 1) “People” as a key success factor when providing TMH services; 2) Automatization and integration of TMH care patient data into an EMR; and 3) Maximizing the synergy of in-person and TMH care.

Evidence supporting the implementation process of telehealth services is limited due to the lack of proven sustainability [40], especially for TMH services. However, the success of a TMH service cannot depend only on technological improvements. For example, in the TMH clinic, there was a historical upgrade of the videoconferencing software to individual desktops (an improvement over the previous fixed videoconferencing system), but the organizational structure, management and leadership supporting the delivery of the services remain ongoing concerns. In our study, an important element that defines the organizational culture of the TMH clinic is the factor of “people”, in terms of leadership and interactions. One study indicates that strong leadership and a good collaboration between the program and the psychiatrists are key elements to continued success [41].

All participants feel a strong sense of team cohesion, and one that is committed to their mandate in serving the Indigenous peoples of Northern Quebec. In addition, a culture of rapid communication, facilitated by the physical layout of the TMH clinic, enhances the sense of teamwork and collaboration, which are key factors for success in TMH [42] and other telehealth settings [43]. To develop a sustainable TMH program, there is a need for centralized support staff who provide administrative, logistic and technical support [44, 45] and attempting to implement TMH without properly trained personnel risks programmatic failure [46, 47]. When technical and scheduling conflicts arise, administrative support staff should readily resolve those issues and maintain the overall functioning of the TMH service [41]. Nonetheless, the views of these critical support staff are undervalued, and in one study [10], researchers aggregated the views of healthcare professionals, managers, an evaluator and an administrative assistant under the umbrella term ‘clinicians’, without providing explicit feedback from the support staff. Another critical element for success is the organizational workflow, such as the management of medical records. The variety of documentation systems reduces the efficiency of the clinic, creating a sense of frustration. Challenges related to documentation are not unique to our study; the inability to integrate telehealth-generated patient data into the EMR is a recurrent issue [48], and evidence exists that EMR solutions, ideally with multimedia capabilities, are necessary to telehealth success [46].

The monthly in-person visits by the TMH psychiatrists to the communities may be used as opportunities to pass on knowledge to the local healthcare providers. According to our findings, psychiatrists enjoy visiting and working with the Northern clinic staff as they develop the “insider” perspective through in-person visits, while maintaining the “outsider” perspective through TMH. The need for both an insider and outsider identity is also supported in other studies outside of the Indigenous context [23, 49], and is a key finding of this study. The reason for the universal appreciation of the synergistic combination of modalities may be due to the inherent complexities involved in Indigenous mental health care, such as the historical trauma, institutionalization, colonization and cultural differences [50]. As for the Indigenous patients, they believe that it is essential for providers to have a good understanding of the local land and community in order to deliver effective TMH care [18]. Additionally, providers serving Indigenous patients have noted that TMH can be impersonal and conflict with Indigenous cultural expectations [14], further highlighting the importance of in-person consultations. Nonetheless, the inherent privacy and interpersonal distance that are required in Indigenous mental health [14] may be facilitated by technologies such as telehealth [51]. Moreover, our study is the first to deeply explore the delicate balance between the “insider” and “outsider” roles of Indigenous mental health care.

### Limitations and future research

The findings of this study, like all qualitative research, are linked to a certain context. Therefore, clinicians, managers and policy makers, who aim to integrate TMH services for Indigenous peoples, should evaluate the applicability of the results to their specific milieus. For example, this study is conducted in a clinical work environment that must follow the policies of a university-hospital network and that uses a documentation system that is mainly paper-based, limiting the transferability of findings to other TMH systems. Another issue of transferability is the fact that other TMH clinics across Canada can potentially be very heterogenous with regards to staffing, communities served and technology. This is especially relevant, as different Indigenous communities have different needs and challenges. Furthermore, due to confidentiality reasons, the research team does not know the exact number of patients being served per TMH psychiatrist, important information which could have influenced the interpretation and transferability of the results. Relatedly, the results of the study are mostly from the perspective of the psychiatrists, and the limited amount of information coming from support staff may affect the applicability of these findings to other contexts.

Undoubtedly, more research, qualitative as well as quantitative, is needed on the functioning of TMH teams and the separate but important roles of each team member. In addition, the attitudes that Indigenous peoples have towards TMH and its effectiveness in terms of accessibility and clinical outcomes should be studied. This would address a significant limitation of this study, as there are no TMH staff in this study identifying as Indigenous. Consequently, results focus on the practical implications of TMH, and the results and interpretations may not be representative of the realities being faced by Indigenous peoples. Therefore, studies on the experiences of Indigenous patients, healthcare workers and support staff are desperately needed, and conducted in an appropriate fashion, such as using the CONSIDER Indigenous research reporting criteria [52]. Lastly, the Inuit and Cree of Northern Quebec are two different Indigenous peoples and have experienced different forms of colonialism. Thus, future studies should investigate the experiences of Inuit and Cree separately.

## Conclusion

To conclude, the study findings provide clinical and organizational insights of a TMH staff comprised of psychiatrists and support staff. Their exceptional team dynamic allows them to deliver appropriate TMH care to the Indigenous Cree and Inuit peoples of Northern Quebec. The results underscore the importance of combining both in-person and TMH care for Indigenous mental health. Furthermore, there is need for the adoption of a comprehensive EMR, as data generated by TMH is difficult to integrate. This may increase the communication and collaboration with the Northern clinics as well as the efficiency of the TMH clinic and professional satisfaction of its staff. It is hoped that these study findings may be used to directly improve the organization and delivery of mental health care in Northern Quebec, Canada.

## Supplementary Information

**Additional file 1.** Interview Guide

## Data Availability

Due to the explicit assurances of the authors to participants that their confidentiality would be respected, all research data will not be available.

## Abbreviations

EMR: Electronic Medical Record
GP: General Practitioner
TMH: Telemental Health
