# Supplementary material for: Experiences of psychiatrists and support staff providing telemental health services to Indigenous peoples of Northern Quebec
Source: BMC Health Serv Res. 2021 Jan 23;21:85. doi: 10.1186/s12913-021-06072-5 (PMC7825224; doi:10.1186/s12913-021-06072-5)
Supplement: Supplementary file 1 — Additional file 1. Interview Guide [file 12913_2021_6072_MOESM1_ESM.docx]

**Interview Guide**

1. Tell me about your role within the Telemental health clinic.
   - How did you get involved in TMH, and what drew you to this current position?
   - What is in your opinion the purpose of the TMH?
   - Do you have other responsibilities?
2. Can you describe a typical working day?
   - What issues do you typically experience during your work in the TMH clinic? Can you give some examples?
   - Are the majority of your activities planned or unplanned? (e.g., how often are you called for unscheduled events?)
3. I have also learned that the weekly rounds are a routine feature for TMH clinic staff. What kind of input do you provide during those weekly rounds?
   - In your opinion, what is the purpose of these weekly Thursday rounds?
4. Can you describe the relationship between yourself and the other TMH staff?
   - How do you work together with them? What is the main method of communication within the TMH clinic?
   - What issues do you typically encounter and how/who do you call for support?
   - Besides the clinic staff, who else do you regularly work with? Can you describe to me how you work together?
5. Now I would like to talk about the videoconferencing sessions here in the clinic. Can you explain how a TMH consultation is organized?
6. In your opinion, what are the challenges you currently encounter when organizing and delivering a TMH videoconferencing consultation?
   - Conversely, what are, in your opinion, the advantages of videoconferencing compared to face-to-face interactions?
   - Do you believe that this videoconferencing technology could influence the therapeutic alliance? If so, can you elaborate?
   - Can you describe to me the process of what happens when there is a technical problem? How was it resolved?
7. Has an emergency ever occurred during a videoconferencing session before? Can you briefly describe to me what happened and how you dealt with it?
   - If not, how prepared do you think you are to deal with these potential emergencies?
8. I also know that you do both initial visits and follow-up visits by videoconferencing.
   - Is there a difference in the way you approach these two types of videoconferencing sessions? Can you explain?
   - Do you believe that it would be beneficial for all initial visits to be done in-person up North? Why or why not?
   - While doing the videoconferencing follow-ups, how do you know if a patient has improved?
9. According to the literature, missed videoconferencing consultations by patients are a significant issue.

- What do you do when a patient does not show up for their consultation?
- What do you think may be the reasons for patients to miss their videoconferencing appointment?

1. Tell me about the scheduling system for videoconferences. Is there anything that can be improved?
2. Next, I would like to talk about your experiences working with the Northern staff.
   - What is your experience communicating with local frontline workers in the Northern communities?
   - Do you go in-person to the Northern communities? If so, which communities do you go to typically? Do you always stay in the same community?
     - If not, why do you think that the psychiatrists travel monthly up North?
     - What do you think are the benefits of being in the same community?
   - Are there any sort of work dynamics that are unique to working with the Northern team, in terms of culture or mannerisms?
3. I have also learned that there are a few Northern patients who are hospitalized on the inpatient unit here at the Mental Health University Hospital.
   - Do you work on the inpatient unit or with the inpatient staff? If so, what is it like?
     - What are the differences in working with the inpatient staff, compared to the TMH clinic staff?
   - Upon discharge, do you know which patients would need the videoconferencing follow-ups and which ones will not? How could you tell?
4. Tell me about the documentation system that is currently being used.
   - Is there a specific software you are using?
     - Do you use a specific software to communicate among members of the TMH clinic?
     - If not, would it be interesting to have such a system?
   - Is the documentation system universal, or is it only used in a certain context (e.g., only up North or only in the inpatient unit)?
   - What can be improved? What will be the advantages and disadvantages of implementing an electronic health record?
5. In your opinion, what are your recommendations to improve the TMH services?
6. What do you think is the future of telehealth, mhealth for delivering TMH services?
   - What are some future developments that you would like to see in the field of TMH in the future?
     - Do you believe that the TMH clinic can adopt this in the future? Why or why not?
7. Is there anything else that you would like to talk about?

Thanks!
